# Biological Maturation Is Associated with Single-Leg Jump Performance, but Not with the Magnitude of Inter-Limb Asymmetry

**DOI:** 10.3390/sports14040163

**Published:** 2026-04-17

**Authors:** Gennaro Boccia, Giulia Paurini, Daniele Villano, Roberto Marocco, Alexandru Nicolae Ungureanu, Luca Beratto, Paolo Riccardo Brustio, Alberto Rainoldi, Corrado Lupo

**Affiliations:** 1 Department of Clinical and Biological Sciences, University of Turin, 10126 Turin, Italy; gennaro.boccia@unito.it (G.B.); paoloriccardo.brustio@unito.it (P.R.B.); 2 Neuromuscular Function Research Group, School of Exercise and Sport Science, University of Turin, 10126 Turin, Italy; giulia.paurini@edu.unito.it (G.P.); daniele.villano@edu.unito.it (D.V.); roberto.marocco@unito.it (R.M.); alexandru.ungureanu@unito.it (A.N.U.); luca.beratto@unito.it (L.B.); alberto.rainoldi@unito.it (A.R.); 3 Department of Life Sciences and Systems Biology, University of Turin, 10123 Turin, Italy; 4 Department of Medical Sciences, University of Turin, 10126 Turin, Italy

**Keywords:** asymmetry, peak height velocity, physical performance, youth basketball, jump test

## Abstract

This study investigated interlimb asymmetries in lower limb performance using both vertical and horizontal jump tests in elite young basketball players. Specifically, it aimed to determine whether (1) unilateral jump performance and (2) the magnitude of interlimb asymmetry differed across maturity groups, whether (3) limb dominance influences performance, and whether (4) asymmetry direction is consistent across tests. One hundred elite male basketball players (U13 to U19) were categorised into three maturational stages: Pre-PHV (*n* = 19), Circa-PHV (*n* = 29), and Post-PHV (*n* = 52). Each athlete performed the following unilateral tests with both the dominant and non-dominant leg: single-leg hop, triple hop for distance, 6 m timed hop, single-leg countermovement jump (SL-CMJ), and single-leg drop jump (SL-DJ) from a 30 cm box. The Bilateral Strength Asymmetry (BSA) index was computed for each test. All tests showed significant differences between Pre-PHV and Circa-PHV groups (*p* < 0.001), whereas only the 6 m timed hop differed between Circa-PHV and Post-PHV (*p* < 0.01). BSA did not differ significantly across maturation stages in any test, except for the single-leg hop. Agreement in asymmetry direction between test pairs was slight to fair (kappa ≤ 0.29). BSA values remained largely stable across maturational stages, suggesting that interlimb asymmetries are established before PHV, likely during childhood. Limb dominance did not affect jump performance, and asymmetry direction varied between tests, confirming they are not interchangeable.

## 1. Introduction

Interlimb asymmetries refer to differences between limbs in structure, function, or performance. Such asymmetries are common in humans and may arise from biological variability, task-specific demands, training history, or limb dominance [1]. However, their practical relevance remains unclear, as their associations with performance, injury risk, and maturation appear remains equivocal [1]. A common misconception is that symmetry is advantageous for sports performance. While some studies report detrimental effects of asymmetries [2], evidence remains inconsistent [3,4], and many athletes achieve high performance despite substantial interlimb differences [1]. Moreover, asymmetry is often misinterpreted as a cause rather than a consequence of training or injury, although prospective evidence linking asymmetry to injury risk is weak and inconsistent [5,6].

Limb dominance, often cited as a prime example of asymmetry, highlights the role of lateralization in human movement. According to the dynamic-dominance model [7,8], the dominant limb is specialised for predictive control and execution under stable condition, whereas the non-dominant limb stabilises movement under variable mechanical conditions. Conversely, dominance does not appear to confer consistent advantages in strength or power performance: two meta-analyses found no influence of lower-limb dominance on isometric strength or dynamic jump tests [9,10].

Interlimb asymmetries are also highly task-specific, as they manifest differently depending on the metric or test applied [11]. The direction of asymmetry is rarely consistent across assessments, leading to poor agreement in classifying individuals as symmetric or asymmetric [12,13,14,15,16]. For instance Bishop [17] found that agreement between jump tests was low, with individual limbs alternately favoured depending on the task. Consequently, the recent literature emphasises adopting an individual approach and employing multiple assessments to capture the multifaceted nature of asymmetry [11,18,19]. The influence of sport-specific demands further complicates interpretation. For example, sports such as tennis or fencing involve repetitive unilateral loading, whereas basketball features primarily bilateral movements—jumping, landing, sprinting, and change of direction—performed with both legs [20,21]. Thus, basketball provides an ideal model for examining how maturation affects asymmetry without the confounding influence of chronic unilateral overload.

Despite extensive research, little is known about how interlimb asymmetries evolve across maturation in youth athletes. As children transition into adolescence, they undergo substantial changes in their musculoskeletal systems, neuromuscular control, and biomechanical characteristics, all of which may influence the expression of interlimb asymmetry [22]. In particular, the rapid increases in limb length and body mass that occur around peak height velocity (PHV) may temporarily alter segmental inertia and challenge motor coordination, potentially affecting the control and distribution of force production between limbs. During periods of rapid growth, adolescents may experience transient imbalances in strength and coordination between their limbs while increasing their performances [23,24]. These imbalances may exacerbate pre-existing asymmetries or lead to the emergence of new ones. Nevertheless, previous studies have generally reported no consistent effect of biological maturation on limb asymmetry in youth athletes, including footballers [25,26,27,28], handball players [29], tennis players [30]. However, some evidence suggests that asymmetries may temporarily increase around PHV [31], while in tennis players, change-of-direction asymmetry magnitude has been shown to decrease with increasing chronological age [32]. Of note, biological maturation (rather than chronological age) is the more appropriate explanatory variable as it better reflects individual developmental timing. Clarifying the common developmental trajectory of asymmetry would help practitioners interpret asymmetry scores more accurately when screening youth populations, particularly around the period of PHV, when young athletes may be more prone to injury [33].

In the present study, we employed a battery of single-leg jump tests that are commonly used in practice and are mechanically distinct enough to examine task-specific agreement. These tests involved different performance demands, namely horizontal versus vertical propulsion and concentric versus reactive muscle actions, thus providing an appropriate framework for the evaluation of task-specific asymmetry. Accordingly, the aims of this study were to determine whether, at a specific time point in a cohort of adolescent basketball players, (1) unilateral jump performance and (2) the magnitude of interlimb asymmetry differed across maturity groups. In addition, we examined (3) whether limb dominance influenced performance and (4) the level of agreement in asymmetry direction across tests. We hypothesised that maturation would significantly affect performance, but not asymmetry magnitude, as maturation was expected to improve the performance of both limbs similarly. We further hypothesised that limb dominance would not influence jump performance and that agreement in asymmetry direction between tests would be low. Finally, as an exploratory objective, we aimed to identify the test most sensitive to detecting interlimb asymmetries, defined as the test exhibiting the greatest asymmetry magnitude.

## 2. Materials and Methods

### 2.1. Subjects

A convenience sample of 100 elite young male basketball players was recruited for the study (≈14 players from each annual age group from U-13 to U-19). The players belong to the youth department of a basketball club that competes in the Italian Serie A2 (i.e., the second national division). The training age was not recorded, nor the volume of jumping drills in their training programs, even if that information might help in interpreting the results of the present study. The same two practitioners administrated all the tests. An a priori power analysis indicated that a total sample size of 100 participants would be sufficient to detect a medium-to-large effect (Cohen’s f = 0.3) with 80% power and an alpha level of 0.05 in a one-way ANOVA with three groups according to G*power (version 3.1.9.6) [34,35]. We assumed balanced group allocation and we did not account for potential imbalance between maturation group. Inclusion criteria required participants to be youth basketball players who trained and competed regularly for at least five hours per week and were willing to participate. Exclusion criteria included any lower-limb injury within the previous 12 months or any chronic condition that could affect limb asymmetry (e.g., neurological disorders or structural asymmetries identified by medical staff). Injury history was collected through athlete self-report and verified with the team staff. However, none of the contacted athletes were excluded on this basis. Testing was conducted during the competitive season to ensure that the results reflected the athletes’ peak physical condition. All tests were conducted on the same day in a randomised order, with individual counterbalanced randomization. Players were required to attend a familiarisation session immediately before testing to understand the test protocols. During the familiarisation session, athletes were allowed to practice the tests, until they demonstrated correct execution according to the investigator’s instructions. Practice attempts were limited to ensuring task comprehension, limiting as much as possible learning effects. All athletes were tested during regular training periods and they were asked to continue with their normal dietary, coffee intake, and sleeping habits before the test session. All participants and their parents were informed about the testing procedure and provided written informed consent before participating in the experiments.

The sample was divided into three groups according to maturational stage: >1 year before PHV (Pre-PHV), ±1 year from PHV (Circa-PHV) and >1 year after PHV (Post-PHV). The predicted maturity offset was estimated, defined as the time before or after PHV. The Pre-PHV group was defined as the period one year before the age of PHV, the Circa-PHV group as ±1 year from PHV, and the Post-PHV group as more than one year after PHV. The following four variables were recorded for each subject to calculate biological age: chronological age (test day minus birthday), stature, sitting height and body mass. Leg length was calculated as stature minus sitting height. Stature and sitting height were measured using a digital stadiometer (Seca 242, Seca, Hamburg, Germany), while body mass was measured using a digital scale (Seca 769, Seca, Hamburg, Germany). Players were evaluated while wearing only undergarments and barefoot. These variables were used to calculate the PHV offset using the formula proposed by Mirwald et al. [36].


Maturity Offset for boys (years) = −9.236 + (0.0002708 × leg length ×
sitting height) + (−0.001663 × age × leg length) + (0.007216 × age × sitting
height) + (0.02292 × mass by stature ratio × 100)

Percentage of predicted adult height (PAH) was also calculated as the average of the self-reported height of the two parents increased by 6.5 cm [37] to furthermore describe the three groups in terms of biological maturation status and proximity to their expected final height, but it did not contribute to group allocation [38].

### 2.2. Procedures

At the beginning of the experimental session, lower-limb dominance was assessed using the inventory of dominance based on three commonly used functional tasks: the preferred leg to kick a ball, to step onto a platform, and to regain balance after a perturbation. This approach is widely adopted and considered a valid proxy for determining limb dominance in both research and clinical settings [39]. Afterwards, all players performed a standardised 10 min warm up composed by jog, and sport specific movement preparation performed at progressive intensity. Subsequently, participants performed a test battery consisting of a series of unilateral functional tests in a randomised order. Each test was performed two times for each limb, the dominant and non-dominant one, with the order randomised. The best attempt was kept for each limb for following analysis. Participants are instructed to give their maximal effort while maintaining control and proper landing technique. Rest periods of 30 s were provided between trials to minimise fatigue. The tests included in the battery are following reported and includes both horizontal and vertical single-leg jumps. Participants were not allowed to swing their arms during the execution of the tests so that their hands were kept in touch with the hips. The intraclass correlation coefficients (ICC) are reported for each test at the end of the description.

1. Single-Leg Countermovement Jump (SL-CMJ)

The participant stands on one leg on a marked area and performs a rapid downward movement followed immediately by a vertical jump. The goal is to achieve maximal vertical height. The participant must land on the same leg and maintain balance. The Optojump™ system (Microgate, Bolzano, Italy) was used to measure flight time. This system consists of two parallel bars: a transmitter with 96 light-emitting diodes positioned 0.003 m above the ground and a receiver. When a foot interrupts the light during a jump, the system records with 1 ms precision, measuring ground contact time as the total interruption time and flight time as the time between interruptions. For this study, the Optojump™ bars were positioned one metre apart (ICC = 0.97) [40].

2. Single-Leg Drop Jump from a 30 cm Box (SL-Drop jump)

The participant stands on one leg atop a 30 cm high box, then drops down and immediately performs a maximal vertical jump upon ground contact, minimising ground contact time while maximising jump height. The participant should aim to land on the same leg while maintaining postural control. As mentioned before, jump height was measured via flight time formula with Optojump™ system (ICC = 0.91) [40].

3. Single-Leg Hop for Distance (Single-leg hop)

The participant stands on one leg behind a marked starting line and performs a maximal forward hop, aiming to cover the greatest possible horizontal distance while maintaining balance on landing. The participant must land on the same leg and hold the landing position for at least two seconds without placing the opposite foot on the ground or losing balance. The distance from the toe at take-off to the heel at landing is measured (ICC = 0.94) [41].

4. Triple Hop for Distance (Triple hop)

Starting from the same position as the single-leg hop, the participant performs three consecutive maximal forward hops on the same leg. The goal is to cover as much horizontal distance as possible while maintaining control and balance. The test ends when the third landing is completed and stabilised. The total distance covered from the starting line to the final landing point is measured (ICC = 0.97) [41].

5. 6-Metre Timed Hop (6 m timed hop)

The participant starts from a stationary position on one leg behind a marked line and hops forward on the same leg as fast as possible to cover a 6 m distance. The test ends when the foot crosses the 6 m mark. Time, in seconds, is recorded using timing gates (Witty Timing System, Microgate, Bolzano, Italy), with the first gate positioned at the starting line and the second gate at the 6 m mark. Gates were placed at approximately hip height (~1 m) to ensure consistent detection of the athlete’s centre-of-mass region (hip level). Timing was triggered by the athlete’s initial forward movement crossing the first gate and stopped when the same anatomical region (hip level) crossed the second gate. As with the other tests, participants were not allowed to swing their arms while performing the task; consequently, their hands remained in contact with their hips. Loss of balance, touching the ground with the opposite foot, or excessive forward trunk inclination leading to premature gate triggering invalidated the trial (ICC = 0.87) [42].

### 2.3. Statistical Analysis

Descriptive values of the dependent variables are reported as mean ± SD. Three separate MANOVAs were initially conducted with maturity offset category (i.e., Pre-PHV, Circa-PHV, Post-PHV) as the between-subject factor, and anthropometric measures, single-limb performance variables, and BSA values as dependent variables, in order to assess the overall effect of maturation on each set of outcomes. Subsequently, separate one-way ANOVAs were performed to allow a clearer interpretation of each anthropometric, performance, and asymmetry variable. To address the exploratory aim, a repeated-measures ANOVA was conducted with test as the within-subject factor and BSA as the dependent variable.

Before running the ANOVAs, normality was assessed using the Shapiro–Wilk test and homogeneity of variance using Levene’s test. When homogeneity of variance was violated in the between-group ANOVAs, Welch’s ANOVA was applied to account for unequal variances and unequal group sizes. For pairwise comparisons in these cases, the Games–Howell procedure was used. To account for the skewed distribution of the asymmetry data, BSA values were square-root transformed prior to analysis, which resolved deviations from normality. In the repeated-measures ANOVA, sphericity was assessed using Mauchly’s test, and when this assumption was violated, the Greenhouse–Geisser correction was applied. In all other cases, post hoc comparisons were adjusted using the Bonferroni method. The alpha level was set at 0.05. Effect sizes were interpreted using partial eta squared (η^2^_p_), with thresholds of 0.03, 0.08, and 0.20 corresponding to small, medium, and large effects, respectively.

The index of asymmetry was calculated using the BSA [43], which gives the same quantitative information of the *percentage difference method* proposed in [44], using the highest value collected between the two limbs for each metric considered to identify the Stronger Limb, and vice versa:

$$\mathrm{BSA}\;\left[\%\right]=\;\frac{\mathrm{Stronger}\;\mathrm{Limb}-\;\mathrm{Weaker}\;\mathrm{Limb}}{\mathrm{Stronger}\;\mathrm{Limb}}\times 100$$

The BSA has been extensively reported to be a more straightforward and interpretable metric compared to the other available asymmetry index because it use the stronger side as reference [43,44]. We also calculated the smallest worthwhile change (SWC, 0.2 × pooled SD) [45] to interpret interlimb difference that exceeded this threshold as a true difference [46]. Participants were considered symmetric when the interlimb difference was less than SWC [13]. Otherwise, they were considered asymmetric, favouring either the dominant or the non-dominant side. Then, to answer the fourth experimental question Kappa coefficients were calculated to determine the levels of agreement for the direction of asymmetry among tests at the individual level [12]. Cohen’s kappa quantifies the level of agreement between two methods beyond that expected by chance alone [47]. Because asymmetry direction was classified into three ordered categories (i.e., favouring the dominant limb, symmetry, and favouring the non-dominant limb), a linearly weighted kappa was used [48] so that disagreements between adjacent categories were penalised less than disagreements between opposite categories. Kappa values were interpreted as follows [49]: 0.01–0.20 = slight; 0.21–0.40 = fair; 0.41–0.60 = moderate; 0.61–0.80 = substantial; 0.81–0.99 = nearly perfect. High Kappa values would mean that the direction of asymmetry tends to be the same for different muscle groups or metrics. Statistical analyses were performed using JASP software (Version 0.19), with the exception of weighted kappa coefficients, which were calculated using the GraphPad QuickCalcs online kappa calculator (www.graphpad.com/quickcalcs/kappa1/?k=3, accessed on 1 February 2026).

## 3. Results

### 3.1. Anthropometry

The three maturation groups were unbalanced in terms of sample size, with 19 athletes in the Pre-PHV stage, 29 in the Circa-PHV stage, and 52 in the Post-PHV stage. Anthropometric parameters increased significantly across maturation groups, as indicated by the MANOVA (F = 22.1, *p* < 0.001, see Table 1 for descriptive analysis). Follow-up univariate ANOVAs revealed significant differences across maturation status for body mass (F = 64.1; *p* < 0.001; η^2^_p_ = 0.570); stature (F = 137.6; *p* < 0.001; η^2^_p_ = 0.739); sitting height (F = 146.9; *p* < 0.001; η^2^_p_ = 0.752). The same was valid for PAH (F = 125.1; *p* < 0.001; η^2^_p_ = 0.729). The post hoc analysis revealed significant differences in all tests between Pre-PHV and Circa-PHV (all *p* values < 0.001) and between Circa-PHV and Post-PHV (all *p* values < 0.001).

### 3.2. Effects of Maturation on Performance

**Figure 1:**
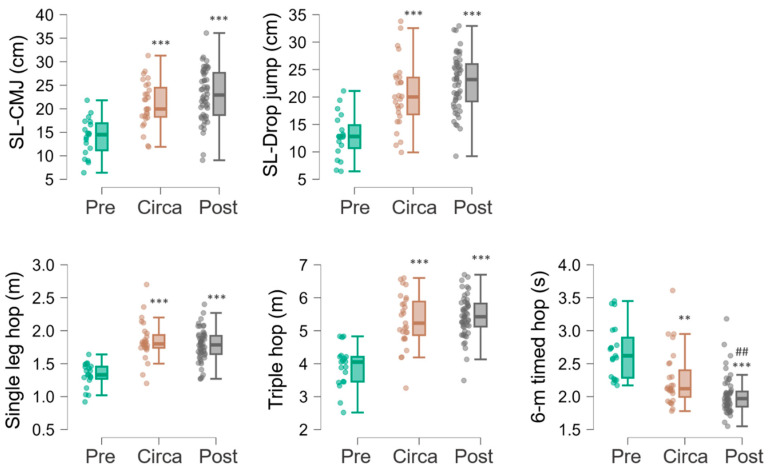
Test performances (averaged between the two limbs) in the three maturation subgroups. SL-CMJ, Single-Leg Countermovement Jump. SL-Drop jump, Single-Leg Drop Jump from a 30 cm Box.; Single-leg hop, Single-Leg Hop for Distance; Triple hop, Triple Hop for Distance; 6 m timed hop, 6-Metre Timed Hop. Differences compared to Pre-PHV are reported as follows: ** *p* ≤ 0.01; *** *p* ≤ 0.001. Difference compared to Circa-PHV is reported as follows: ## *p* ≤ 0.01.

Table 2 shows the performance of dominant and non-dominant limb for each test. The test performances (merged between the two limbs) showed large increase with maturation as showed by MANOVA (F = 8.2, *p* < 0.001, see Figure 1 for descriptive analysis). Follow-up univariate ANOVAs revealed significant differences across maturation status for SL-CMJ (F = 21.5; *p* < 0.001; η^2^_p_ = 0.307); SL-Drop jump (F = 24.0; *p* < 0.001; η^2^_p_ = 0.332); Single-leg hop (F = 25.8; *p* < 0.001; η^2^_p_ = 0.348); Triple hop (F = 32.6; *p* < 0.001; η^2^_p_ = 0.402); 6 m timed hop (F = 17.7; *p* < 0.001; η^2^_p_ = 0.268). The post hoc showed that all tests showed significant difference between Pre-PHV and Circa-PHV (all *p* values < 0.001) and between Pre-PHV and Post-PHV (all *p* values < 0.001). After post hoc correction, the 6 m timed hop was the only test in which a significant difference between Circa-PHV and Post-PHV was observed (*p* < 0.01) (see Figure 1 for the complete post hoc reporting).

### 3.3. Effects of Dominance on Performance

MANOVA did not show overall effect of dominance on performance (F = 0.5, *p* = 0.739), nor interaction between maturation status and dominance (F = 0.3, *p* = 0.954). Follow-up ANOVA results are reported in Table 2. With the exception of single-leg hop in Pre-PHV (post hoc test after correction for multiple comparison), none of the other tests or groups showed any effect of dominance on performance. SWC analysis showed that 24 to 44% of participants were considered symmetric, while the rest were almost homogeneously distributed between favouring the dominant or non-dominant side (Table 2).

### 3.4. Effects of Maturation on BSA

**Figure 2:**
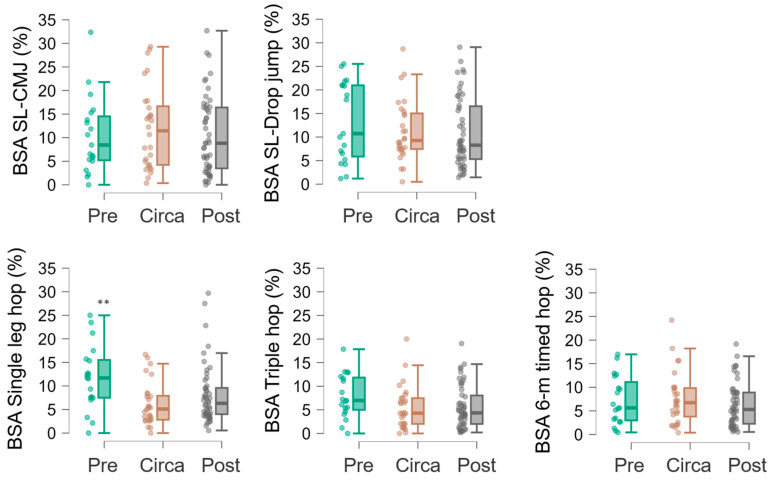
Bilateral Strength Asymmetry (BSA, %) reported in each test in relation to the three maturation subgroups. Difference compared to Circa-PHV is reported as follows: ** *p* ≤ 0.01.

Figure 2 shows the BSA index for each test, broken down by group. MANOVA did not show overall difference across maturation status (F = 1.3, *p* = 0.186). Successive ANOVAs did not show differences across maturation status for BSA index in SL-CMJ (*p* = 0.707), SL-Drop jump (*p* = 0.559), Triple hop (*p* = 0.146), or 6 m timed hop (*p* = 0.523). Conversely, maturation had a medium effect on the single-leg hop (F = 4.7; *p* = 0.014; η^2^_p_ = 0.089), showing that the BSA index was larger in the Pre-PHV group than in the Circa-PHV and Post-PHV groups.

### 3.5. Agreement

Kappa coefficients and exact agreement (i.e., the frequency of two tests indicating the same outcome in terms of asymmetry direction) are reported in descending order in Table 3. All agreements were between slight and fair. The 6 m timed hop showed negative kappa values with all other tests.

### 3.6. Explorative Analysis

**Figure 3:**
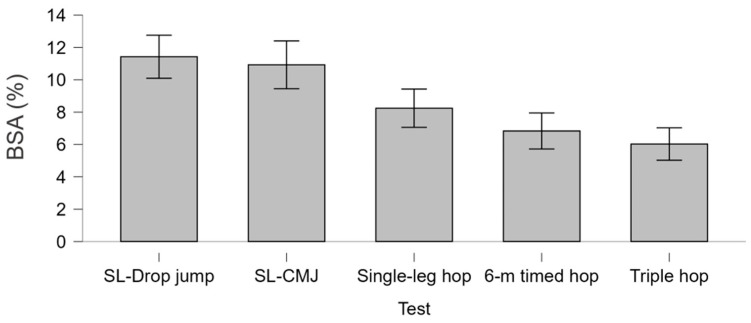
Average BSA (bilateral strength asymmetry) index (%) for each pair of tests in descending order. SL-CMJ, Single-Leg Countermovement Jump. SL-Drop jump, Single-Leg Drop Jump from a 30 cm Box.; Single-leg hop, Single-Leg Hop for Distance; Triple hop, Triple Hop for Distance; 6 m timed hop, 6-Metre Timed Hop.

Figure 3 reports the average BSA index for each pair of tests in descending order (F = 15.0; *p* < 0.001; η^2^_p_ = 0.136). The tests that highlighted the highest magnitude of asymmetry were the SL-Drop jump and SL-CMJ (BSA ≈ 11%), while the 6 m timed hop and Triple hop showed the smallest magnitude of asymmetry on average (BSA ≈ 5%).

## 4. Discussion

We investigated interlimb asymmetry in lower limb performance using both vertical and horizontal jump tests in a sample of 100 elite young basketball players across different stages of maturation. Our findings revealed that: (1) performance outcomes differed according to maturation status, with significant differences observed between Pre-PHV and Circa-PHV, whereas differences between Circa-PHV and Post-PHV were less evident and limited to specific tests; (2) asymmetry magnitude (BSA) did not differ across maturation groups; (3) limb dominance had no significant effect on performance outcomes; and (4) the agreement in asymmetry measures across different tests was generally low (all Kappa < 0.3), suggesting that asymmetries are task-specific.

There were consistent differences in anthropometric characteristics across maturation groups: body mass, stature, and sitting height showed significant differences between Pre-PHV and Circa-PHV, as well as between Circa-PHV and Post-PHV (Table 1). This pattern, however, was not observed for performance outcomes, highlighting the complexity of the relationship and suggesting that multiple factors may be involved. For instance, a previous study [50] suggested that anthropometric increases may contribute to variation in physical performance throughout maturation, with different trends observed between boys and girls. Moreover, maturity has been reported to be strongly associated with sprint performance in English male academy soccer players, whereas the same pattern was not confirmed for the CMJ [51], further highlighting the complexity of the relationship and the possible influence of task characteristics. By contrast, findings in young volleyball players showed that maturity status was strongly and positively associated with jump performance regardless of sex [52]. In our study, performance differences were evident only between the Pre-PHV and Circa-PHV groups, whereas only one out of five tests showed differences between Circa-PHV and Post-PHV groups (Figure 1). Since single-limb jump tests require athletes to displace their body mass during the propulsive phase, it is plausible that the limited differences observed between Circa-PHV and Post-PHV may be partly related to concurrent anthropometric changes during this stage. However, as no statistical adjustment for anthropometry was performed and no mechanistic variables were directly examined, this interpretation remains speculative. The 6 m timed hop was the only test that continued to show improvement after PHV. One possible explanation is that this task may be influenced differently by maturation-related anthropometric changes than the other jump tests, although this was not directly examined in the present study. Therefore, although anthropometric development and physical performance are closely related during adolescence, the observed differences across maturation groups should be interpreted cautiously and in light of the concurrent anthropometric changes.

We found that absolute levels of interlimb asymmetry (i.e., BSA) did not vary across maturation stages in any analysed tests (Table 2 and Figure 2). BSA values remained remarkably consistent across the Pre-PHV, Circa-PHV, and Post-PHV groups. However, given the cross-sectional design of the present study, it cannot be concluded that maturation status has no effect on asymmetry; rather, our findings indicate that no differences were observed among the maturity groups examined in this sample. This trend is consistent with findings from previous studies in elite male youth soccer players [22,35,53] although different results were reported in a study on young tennis players, in which the circa-PHV group showed greater asymmetry scores than the other groups [31]. Therefore, these findings may suggest that the neuromuscular and biomechanical changes often associated with the PHV period—such as temporary alterations in coordination or movement execution—do not necessarily translate into greater asymmetries between limbs in the present sample. In other words, although adolescence and the PHV phase may affect overall motor control [54,55], our findings do not indicate a consistent preferential effect on one side of the body over the other.

Dominance did not affect performance in any test or maturation group, except for the single-leg hop in the Pre-PHV phase (Table 2). This finding is consistent with previous meta-analyses showing that lower limb dominance does not influence muscle strength or jump performance outcomes [9,10]. The absence of an effect of limb dominance may indicate that other factors, such as technique, coordination, or training background, are also relevant in determining performance in single-limb tests. The absence of an interaction between maturation and dominance indicates that the role of limb dominance did not change across maturation stages, at least in proximity to PHV. From a motor control perspective, single-leg tests are performed under stable conditions (i.e., without external perturbations), which may favour the dominant limb due to habitual use and greater control [7]. At the same time, these tasks require body stabilisation and impedance control, which might favour the non-dominant limb in certain individuals [8]. Therefore, it may be speculated that the nature of single-limb testing could confer different task-related advantages to both limbs, potentially contributing to the absence of significant differences. The better performance observed in the single-leg hop in the Pre-PHV group may reflect task-specific factors not directly assessed in the present study, such as movement strategy or previous exposure to similar tasks. In all other tests and across maturation groups, no meaningful performance differences were observed between the dominant and non-dominant limbs. This finding is further supported by the distribution of limb preference, which showed that athletes performing better on the dominant or non-dominant side were evenly distributed, with approximately 30–40% favouring either side across all tests and maturation stages (Table 2).

Asymmetries rarely favoured the same side across different tests, indicating that asymmetry direction was inconsistent at the individual level. Except for a few test pairs, most Kappa coefficients were below 0.2, indicating poor direction agreement between tests (Table 3). Kappa values close to 0 suggest that any agreement observed was likely due to chance, despite acknowledging that kappa can underestimate agreement when category prevalence is uneven. Therefore, if one metric favoured the dominant limb in a specific test, this did not necessarily occur in another test. This suggests that participants do not exhibit a consistently more performant limb across all tasks. Instead, the findings support the view that inter-limb differences are task-specific and may vary according to the motor and mechanical demands of each test. The task-specific nature of inter-limb differences has been clearly demonstrated in previous studies by Bishop and colleagues [12,17,56]. Nevertheless, a subset of tests did show slightly higher agreement. The single-leg drop jump showed fair agreement with the triple hop, single-leg hop, and single-leg countermovement jump (Kappa values ranging from 0.230 to 0.251) [57]. The single-leg hop showed fair agreement with the single-leg CMJ and triple hop (Kappa values between 0.276 and 0.291). However, even within these pairs of tests, the exact agreement reported in Table 3 indicates that in fewer than 50% of cases both tests favoured the same limb. This confirms that these tests are not interchangeable. Notably, this also applies to tests included in the commonly used hop test battery (single-leg hop, triple hop, 6 m timed hop) [58].

In the exploratory analysis, the vertical jumps—specifically the SL-Drop jump and SL-CMJ—revealed the greatest interlimb asymmetry (Figure 3). This is consistent with evidence showing that male athletes recovering from ACLR may achieve symmetry in horizontal hop and strength tests while still presenting deficits in vertical performance. In particular, persistent asymmetries have been reported in vertical jump height, reactive strength index, concentric impulse, and landing force, especially during SL-Drop jump and SL-CMJ [59,60,61]. Therefore, these tests may be more sensitive than horizontal hop distance for detecting interlimb asymmetries in athletes returning to sport after ACLR [62]. However, the ACLR examples here are used as a conceptual reference for test sensitivity, not as a direct comparison with our study which involved only healthy participants.

Although this study applied various measures and statistical approaches to investigate how maturity and interlimb asymmetry influence jump performance in young elite basketball players, the findings cannot be readily generalised to populations with different ages, competitive levels, or sporting backgrounds. Indeed, sport-specific demands and individual capabilities may substantially influence the observed outcomes, limiting broader applicability [63]. Nevertheless, the existing literature appears to support a converging trend. For instance, even in sports considered asymmetric for upper limbs, such as volleyball, athletes do not consistently exhibit superior performance in elbow flexor or extensor strength [64]. The current sample, composed of pre-adolescent and adolescent athletes—a group known for its physiological heterogeneity—remains a valuable population for such investigations. However, the cross-sectional design of the present study does not allow direct causal inferences regarding the relationship between maturation and asymmetry levels. Longitudinal studies are needed to confirm and extend the trends observed here. Moreover, differences in group size may have influenced the statistical power and may limit the balance of comparisons across maturity categories. This distribution reflects the practical characteristics of the available athletic sample, but should nevertheless be considered when interpreting the findings. As methodological notes, the estimation of jump height based on flight-time-derived calculations may introduce a degree of measurement error. Furthermore, pre-test conditions such as caffeine intake, sleep, and recent training load were not standardised. Finally, different formulas exist to estimate PHV using anthropometric data [38]; for example, Moore et al. [65] proposed an improved version of the original Mirwald equation [36]. However, when we applied the Moore formulas, only 3 out of 100 players changed their maturity classification. Statistical analyses conducted using this alternative stratification yielded results very similar to those presented herein (all η^2^_p_ differences < 0.02), and the main findings remained unchanged. However, potential error associated with maturity offset estimation may remain.

## 5. Conclusions

In conclusion, this study showed that performance differed across maturation stages in young elite basketball players, whereas the degree of interlimb asymmetry remained largely unchanged across the groups examined. Indeed, BSA values were consistent across maturational groups, indicating that no significant differences in absolute interlimb asymmetry were observed in this sample. Additionally, limb dominance did not significantly affect performance across most tests and maturation groups. Finally, the agreement between asymmetry measures across different tests was generally low, supporting the notion that interlimb asymmetry may be task-specific. Vertical jumps exhibited greater asymmetries than horizontal jumps, suggesting that these tasks may be more sensitive for identifying interlimb performance differences.

From a practical perspective, the absence of differences in asymmetry magnitude across maturation groups suggests that practitioners should not assume that these imbalances differ systematically according to maturation stage alone, and should instead continue to monitor them through targeted assessment and training. Similarly, as limb dominance did not consistently influence performance, the dominant leg should not be assumed to outperform the non-dominant one. The low agreement between asymmetry measures across different tests further indicates that asymmetries are task-specific, reinforcing the importance of using a battery of assessments rather than relying on a single test. Notably, single-leg countermovement and drop jumps revealed greater asymmetries than horizontal hops, suggesting that vertical jump tests may be useful for detecting inter-limb performance differences.

## Figures and Tables

**Table 1:** Mean and standard deviation of anthropometric measures and predicted adult height across biological maturations subgroups. All participants were males.

	Pre-PHV *n* = 19	Circa-PHV *n* = 29	Post-PHV *n* = 52
Weight (kg)	47.6 ± 9.0	61.7 ± 10.2 ***	75.5 ± 9.2 ***^###^
Stature (cm)	150.7 ± 10.3	169.1 ± 7.2 ***	183.4 ± 6.3 ***^###^
Sitting Height (cm)	75.6 ± 6.9	83.1 ± 3.3 ***	91.2 ± 3.4 ***^###^
Chronological age (yrs)	11.6 ± 1.0	13.9 ± 0.9 ***	16.7 ± 1.2 ***^###^
Predicted adult height (PAH) (%)	83.8 ± 5.1	93.3 ± 3.1 ***	100.3 ± 3.6 ***^###^

PHV, peak height velocity. Differences compared to Pre-PHV are reported as follows: *** *p* ≤ 0.001. Difference compared to Circa-PHV is reported as follows: ^###^ *p* ≤ 0.001.

**Table 2:** Mean and standard deviation of jump test performance for the dominant and non-dominant limbs across biological maturation subgroups. *p*-values refer to the follow-up ANOVA analysis performed for each test. BSA indices and the distribution of participants classified as symmetric or asymmetric are also reported.

		Dominant	Non-Dominant	BSA (%)	Participant Favouring Non-Dominant/Symmetric/Dominant Limb (%)	*p* Value for Maturation	*p* Value for Dominance	*p* Value for Interaction
SL-CMJ (cm)	Pre-PHV	14.2 ± 4.4	13.6 ± 3.7	10.4 ± 8.1	33/38/29			0.635
	Circa-PHV	20.8 ± 5.5 ***	20.4 ± 5.2 ***	12.1 ± 8.7		<0.001	0.350	
	Post-PHV	22.9 ± 5.8 ***	23.0 ± 5.5 ***	10.5 ± 8.1				
SL-Drop jump (cm)	Pre-PHV	12.9 ± 4.2	12.8 ± 4.1	13.3 ± 8.5	41/30/29			0.514
	Circa-PHV	20.5 ± 6.4 ***	19.4 ± 6.0 ***	12.3 ± 8.0		<0.001	0.065	
	Post-PHV	22.9 ± 5.2 ***	22.2 ± 5.5 ***	11.5 ± 8.2				
Single-leg hop (m)	Pre-PHV	1.38 ± 0.20	1.26 ± 0.20 ^$^	11.8 ± 6.9	46/24/30			0.172
	Circa-PHV	1.82 ± 0.30 ***	1.80 ± 0.34 ***	6.4 ± 4.7		<0.001	0.011	
	Post-PHV	1.79 ± 0.29 ***	1.76 ± 0.23 ***	8.2 ± 6.2				
Triple hop (m)	Pre-PHV	3.91 ± 0.65	3.87 ± 0.66	7.9 ± 4.7	35/34/31			0.885
	Circa-PHV	5.22 ± 0.93 ***	5.22 ± 0.96 ***	5.8 ± 4.8		<0.001	0.431	
	Post-PHV	5.45 ± 0.67 ***	5.40 ± 0.63 ***	6.0 ± 4.8				
6 m timed hop (s)	Pre-PHV	2.66 ± 0.36	2.70 ± 0.49	7.2 ± 5.2	24/44/32			0.758
	Circa-PHV	2.33 ± 0.62 **	2.35 ± 0.64 **	7.9 ± 5.5		<0.001	0.465	
	Post-PHV	2.02 ± 0.31 ***	2.01 ± 0.30 ***	6.7 ± 6.2				

BSA, Bilateral Asymmetry Index, was calculated as follows: ((Stronger Leg–Weaker Leg)/Stronger Leg) × 100. Participants were classified according to whether their interlimb difference was smaller or greater than the smallest worthwhile change (SWC). Participants were considered symmetric when the interlimb difference was lower than the SWC. The distribution of participants was then expressed as: % of participant asymmetric favouring the non-dominant limb/% of symmetric participants/% of participant asymmetric favouring dominant limb. *p* values of ANOVA are reported in the last three columns. Differences compared to Pre-PHV are reported as follows: ** *p* ≤ 0.01; *** *p* ≤ 0.001. Difference compared to dominant side is reported as follows: $ *p* ≤ 0.05.

**Table 3:** Agreement in asymmetry direction (favouring the dominant) between tests couples.

Test 1	Test 2	Exact Agreement	Kappa
Single-leg hop	SL-CMJ	49%	0.291
Single-leg hop	Triple hop	48%	0.276
SL-Drop jump	Triple hop	48%	0.239
SL-Drop jump	Single-leg hop	48%	0.230
SL-Drop jump	SL-CMJ	47%	0.251
Triple hop	SL-CMJ	36%	0.080
6 m timed hop	SL-Drop jump	31%	−0.026
6 m timed hop	SL-CMJ	30%	−0.100
6 m timed hop	Single-leg hop	28%	−0.102
6 m timed hop	Triple hop	28%	−0.125

## Data Availability

The data presented in this study are available on request from the corresponding author.
